# Depression, health anxiety, and five-year mortality after ST-elevation myocardial infarction: the SEMI-CI cohort

**DOI:** 10.1016/j.ijcrp.2026.200656

**Published:** 2026-05-28

**Authors:** Yasaman Shojaei, Hamidreza Roohafza, Kamran Mehrabani-Zeinabad, Azam Soleimani, Safoura Yazdekhasti, Mohammadamin Sadri, Masoumeh Sadeghi

**Affiliations:** a Isfahan Cardiovascular Research Center, Cardiovascular Research Institute, Isfahan University of Medical Sciences, Isfahan, Iran; b Department of Biostatistics and Epidemiology, School of Health, Isfahan University of Medical Sciences, Isfahan, Iran; c Heart Failure Research Center, Cardiovascular Research Institute, Isfahan University of Medical Sciences, Isfahan, Iran; d Cardiac Rehabilitation Research Center, Cardiovascular Research Institute, Isfahan University of Medical Sciences, Isfahan, Iran

## Abstract

**Background:**

Psychological factors influence prognosis after ST-elevation myocardial infarction (STEMI), yet the long-term prognostic role of depression and health anxiety remains incompletely defined. We investigated whether these factors identify patients at increased mortality risk following STEMI.

**Methods:**

This secondary analysis included 759 patients from the SEMI−CI cohort, admitted between October 2015 and October 2016 and followed for five years. Depression was assessed at baseline using the Patient Health Questionnaire-9 (PHQ-9), and health anxiety was measured using four items from the Diagnostic Criteria for Psychosomatic Research (DCPR). The primary outcome was cardiovascular mortality. Associations were evaluated using Cox proportional hazards models adjusting for demographic, clinical, and cardiovascular risk factors. Health anxiety was treated as an exploratory predictor given limited measurement validity. Sensitivity analyses accounted for available covariates.

**Results:**

Among 759 participants, 189 (24.9%) died from cardiovascular causes over five years. Mortality was higher among patients with depression (60/189, 31.7%) compared to those without depression (115/570, 20.2%). Health anxiety was present in 42 patients (all of them also had depression), 18 (42.9%) of whom died. In fully adjusted Cox models, compared to patients with neither condition, mortality risk was significantly higher in those with only depression (HR 2.45, 95% CI 1.84–3.03) and those with concurrent depression and health anxiety (HR 2.11, 95% CI 0.11–4.09).

**Conclusions:**

In this cohort of STEMI patients, baseline depression was strongly associated with higher five-year cardiovascular mortality, while health anxiety showed a moderate association. These findings highlight the importance of assessing psychological factors as part of post-MI risk stratification, without implying causal relationships.

## 1 Introduction

Cardiovascular diseases (CVDs) continue to be the leading cause of death worldwide, responsible for more than 1.7 million deaths each year [1,2]. Among the various forms of CVD, acute coronary syndromes (ACS), especially ST-elevation myocardial infarction (STEMI), are critical conditions associated with high short- and long-term mortality rates [3]. According to the “American Heart Association (AHA) Guideline for the Management of Patients With Acute Coronary Syndromes” Forecasts suggest that cardiovascular diseases will continue to rank among the leading causes of death worldwide by the year 2030 [4]. The burden of CVDs is increasing rapidly, with the years of life lost due to these diseases expected to nearly double between 2005 and 2025 [5].

Despite major advances in the management of acute coronary syndromes, including contemporary revascularization strategies and optimized pharmacotherapy, long-term outcomes after myocardial infarction remain influenced by a complex interplay of biological (e.g., age and sex), behavioral (e.g., smoking), psychological, and clinical factors [6]. Recent studies have shown that a range of clinical and biological characteristics—including inflammatory responses such as eosinophil levels after primary PCI, comorbidity burden (e.g., osteoporosis), infectious conditions such as COVID-19, participation in cardiac rehabilitation, and demographic disparities—may contribute to variations in post-MI mortality [[7], [8], [9], [10]]. Taken together, these findings highlight the importance of considering a broad range of determinants in post-MI risk assessment and management, among which psychosomatic factors represent one of the most important and potentially modifiable contributors to patient outcomes [[11], [12], [13]].

Psychological conditions such as depression and anxiety have gained attention as important comorbidities in patients with ischemic heart disease, with mounting evidence indicating their negative effect on prognosis [14].

In particular, depression has been linked to increased mortality after myocardial infarction (MI) in many studies. [15,16]. These results vary across different cultural and healthcare settings in such a way that some studies have found no significant association between depression and cardiovascular mortality or even suggested potential protective effects in certain subgroups, highlighting the complexity of this relationship, for instance, patients with mild depressive or anxious symptoms who demonstrate heightened hyper-vigilance, leading to increased medical help-seeking behaviors, earlier hospital presentations, and stricter adherence to frequent clinical evaluations [17,18]. Additionally, studies have shown that variations in sample characteristics, such as age, gender distribution, and other clinical factors, can influence the relationship between post-myocardial infarction mortality rate and depression [19].

Anxiety disorders have also been studied due to their potential to worsen cardiac outcomes [11,20]. In the context of cardiovascular disease, health anxiety must be distinguished from generalized anxiety disorders. While generalized anxiety involves diffuse, broad worries about everyday life, health anxiety is characterized by a persistent, excessive preoccupation with having or acquiring a serious medical illness, often manifesting as the catastrophic misinterpretation of benign physical sensations (e.g., normal heart palpitations) as impending cardiac events [21,22]. The biological plausibility of how such continuous psychological distress impacts cardiac mortality is grounded in several mechanistic rationales. Chronic health anxiety and psychological stress can precipitate autonomic dysregulation, sustained sympathetic nervous system hyperactivity, and hypothalamic-pituitary-adrenal (HPA) axis dysfunction, all of which are known to exacerbate adverse cardiovascular outcomes [23,24]. Meta-analyses have shown that anxiety can raise the risk of adverse cardiac events by up to 36% [25]. Specifically, health anxiety as a form of anxiety that is described by the fear of having, acquiring, or potentially avoiding illness, is one of the factors influencing patients’ outcomes [26]. However, the diversity of anxiety types and the lack of focused research in an Iranian patient cohort limit the broader application of these findings.

Given the high prevalence of depression and anxiety among cardiac patients and the need for better risk stratification in post-MI care, this study aims to evaluate five-year mortality in STEMI patients in relation to their baseline psychological status. Utilizing validated tools, this cohort study assesses whether depression and health anxiety are significant predictors of long-term mortality among middle-income countries patients enrolled in the SEMI−CI registry. The findings may offer valuable insights into the integration of mental health assessments into routine cardiac care and emphasize the role of psychosomatic factors in cardiovascular prognosis.

## 2 Methodology

### 2.1 Study design and setting

This study is a prospective cohort survival analysis based on the fifth-year follow-up of the SEMI−CI study conducted in Isfahan, Iran. This cohort study was conducted and reported in accordance with the Strengthening the Reporting of Observational Studies in Epidemiology (STROBE) guidelines. The SEMI−CI study enrolled patients with ST-elevation myocardial infarction (STEMI) admitted to university-affiliated hospitals between October 2015 and October 2016. The study included four phases: pre-hospital data (symptoms, referral, early treatment), hospital phase (diagnosis, history, procedures, medications), discharge data (final diagnosis and prescriptions), and a 5-year follow-up (medication adherence, MACE, re-hospitalization, mortality) [27]. Patients were followed for five years to assess long-term mortality outcomes. The study protocol was approved by the Ethics Committee of Isfahan University of Medical Sciences (IR.MED.REC.1402.265), and all participants provided written informed consent prior to enrollment.

### 2.2 Participants

Eligible participants were adults with a confirmed diagnosis of STEMI within 24 h of symptom onset. Exclusion criteria included.

1. Incomplete baseline psychological data
2. Loss to follow-up during the 5-year period
3. Withdrawal of consent

Out of 867 initially enrolled patients, 759 participants were included in the current analysis after applying exclusion criteria.

## 3 Data collection

### 3.1 Baseline clinical and demographic data

Information on age, sex, cardiovascular risk factors (diabetes, hypertension, hypercholesterolemia, smoking), and clinical data including Killip class, site of myocardial infarction (MI), number of epicardial territories involved, and ejection fraction (EF) were collected at hospital admission from medical records.

### 3.2 Psychological assessment

• Depression: Assessed at baseline using the Patient Health Questionnaire-9 (PHQ-9). Scores ≥10 were classified as depression, and scores <10 as no depression [28].
• Health Anxiety: Measured using the Diagnostic Criteria for Psychosomatic Research (DCPR), focusing on the health anxiety cluster (4 items). Patients were categorized as having or not having health anxiety. Given the limited number of items, health anxiety was considered exploratory [29].

While robust for diagnostic classification, their application as prognostic indicators for long-term survival in post-MI patients remains exploratory and should be interpreted within this clinical context.

### 3.3 Outcome measurement

The primary outcome was all-cause and cardiovascular mortality over five years. Mortality data were obtained via hospital records, registry data, and telephone interviews with patients or their family members. Date of death was recorded for survival time calculation.

### 3.4 Statistical analysis

• Descriptive statistics: Continuous variables are presented as mean ± standard deviation, categorical variables as counts and percentages.
• Comparison of baseline characteristics: Between groups (alive vs deceased) using Chi-square tests for categorical variables and independent t-tests for continuous variables.
• Survival analysis:
  o Cox proportional hazards regression was used to estimate hazard ratios (HR) and 95% confidence intervals (CI) for the association between depression, health anxiety, and 5-year mortality.
  o Follow-up time was defined as the time from STEMI admission to death or end of study.
  o Proportional hazards assumption was assessed visually and using Schoenfeld residuals.
• Model building:
  o Model 1: Adjusted for demographic characteristics (age, sex)
  o Model 2: Model 1 + past medical history (diabetes, hypertension, smoking, hypercholesterolemia)
  o Model 3 (fully adjusted): Model 2 + clinical factors (EF, Killip class, site of MI, number of epicardial territories)

To prevent multicollinearity arising from the complete overlap between health anxiety and depression in our cohort, we constructed a four-level composite variable for the multivariable Cox proportional hazards model. The categories were defined as: (0) No depression and no health anxiety (used as the reference category) [1], Depression only [2], Health anxiety only, and [3] Both depression and health anxiety. It should be noted that due to the sample distribution, no patients fell into the ‘Health anxiety only’ category (n = 0).

• Missing data: Complete-case analysis was applied; limitations regarding potential bias from missing data are discussed.
• Sensitivity and exploratory analyses: Health anxiety was treated as an exploratory predictor given limited measurement validity. No causal inference was made.

All analyses were performed using SPSS version 24, with two-sided p-values <0.05 considered statistically significant.

## 4 Results

### 4.1 Study population

Of the initial 867 STEMI patients, 759 (87.5%) had complete baseline and follow-up data and were included in the analysis. Compared with excluded patients (n = 108), included patients were slightly younger (mean age 59.8 vs. 61.3 years) and had similar sex distribution (p > 0.05). Baseline characteristics of the cohort are summarized in Table 1.

**Table 1:** Comparison of basic characteristics of risk factors of Study Participants on cardiovascular mortality after 5 years.

Variable		Alive (n = 570)	Dead (n = 189)	p-value
Demographic characteristics				
Age (mean + SD)		58.32 ± 11.51	70.26 ± 12.45	<0.05
Male		488(85.6)	133(70.4)	<0.05
Past medical history				
Smoke		243(42.6)	52(27.5)	<0.05
Diabetes		145(25.4)	75(39.7)	<0.05
HTN		178(31.2)	85(45.0)	<0.05
Hypercholesterolemia		174(30.5)	46(24.3)	0.06
Clinical data				
Killip Class On Admission (CLASS I)		547(96.0)	148(78.3)	<0.05
Site of MI (anterior)		294(51.6)	117(61.9)	0.008
Number of Epicardial Territories^a^	(1)	233(45.9)	45(29.8)	<0.05
	(2)	161(31.7)	46(30.5)	
	(3)	104(20.5)	59(39.1)	
EF <40%		287(58.2)	103(76.9)	<0.05
Psychological factors				
Health anxiety		24 (4.2)	18(9.5)	0.012
Depression		115 (20.2)	60 (31.7)	<0.05

a The number of epicardial territories of 111 patients was missing.

### 4.2 Follow-up and mortality

Over a median follow-up of 5 years (IQR 4.9–5.1), 189 (24.9%) patients experienced cardiovascular mortality. Median survival time among deceased patients was 3.2 years (IQR 1.5–4.5). Patients who died, compared to those who survived, were significantly older (70.26 ± 12.45 vs. 58.32 ± 11.51), more likely to be female (29.6% vs. 14.4%), and had a higher prevalence of diabetes (39.7% vs. 25.4%), hypertension (45.0% vs. 31.2), reduced ejection fraction (76.9% vs. 58.2%), anterior MI (61.9 vs. 51.6%), and higher Killip class (21.7% vs. 4.0%) (all p < 0.05) (Table 1).

### 4.3 Depression and health anxiety

• Depression: More common among deceased patients (31.7% vs. 20.2%, p < 0.05; see Table 1). Depressed patients had higher smoking rates (46.3% vs. 37.3%, p = 0.01) and slightly higher prevalence of hypercholesterolemia (34.3% vs. 29.3%, p = 0.05; see Table 3).

**Table 2:** Comparison of basic characteristics of risk factors of Study Participants based on health anxiety.

Variable		No health anxiety (n = 717)	Health anxiety (n = 42)	p-value
Demographic characteristics				
Age(mean + SD)		60.28 ± 12.6	59.30 ± 10.6	0.64
Male		594(82.8)	36(85.7)	0.37
Past medical history				
Smoke		290(40.5)	21(50.0)	0.12
Diabetes		206(28.7)	12(28.5)	0.51
HTN		252(35.1)	14(33.3)	0.43
Hypercholesterolemia		216(30.1)	19(45.2)	0.03
Clinical data				
Killip Class On Admission (CLASS I)		674(94.0)	39(92.8)	0.38
Site of MI (anterior)		393(54.8)	18(42.8)	0.11
Number of Epicardial Territories	(1)	312(43.5)	23(54.8)	0.56
	(2)	245(34.2)	13(30.9)	
	(3)	160(22.3)	6(14.3)	
EF (<40%)		433(60.4)	25(59.5)	0.57
Psychological factors				
Depression		133(18.5)	42(100)	<0.05

**Table 3:** Comparison of basic characteristics of risk factors of Study Participants based on depression.

Variable		No depression (n = 584)	Depression (n = 175)	p-value
Demographic characteristics				
Age(mean ± SD)		62.12 ± 13.0	57.70 ± 11.54	<0.05
male		469(80.3)	148(84.6)	0.09
Past medical history				
Smoke		218(37.3)	81(46.3)	0.01
Diabetes		168(28.8)	55(31.4)	0.23
HTN		204(34.9)	54(30.8)	0.18
Hypercholesterolemia		169(29.3)	60(34.3)	0.05
Clinical data				
Killip Class On Admission (CLASS I)		531(91.0)	165(94.3)	0.06
Site of MI (anterior)		312(53.4)	99(56.6)	0.25
Number of Epicardial Territories	(1)	235(40.2)	88(50.3)	0.09
	(2)	197(33.7)	50(28.6)	
	(3)	152(26.0)	37(21.1)	
EF (<40%)		370(63.3)	99(56.6)	0.07
Psychological factors				
Health anxiety		0(0)	42(24.0)	<0.05
• Health anxiety: Present in 9.5% of deceased vs. 4.2% of survivors (p = 0.012; see Table 1). All patients with health anxiety also had depression. Hypercholesterolemia was more prevalent among health-anxious patients (45.2% vs. 30.1%, p = 0.03; see Table 2).

### 4.4 Survival analysis

Cox proportional hazards regression was used to examine associations between psychological factors and 5-year cardiovascular mortality. (Table 4).

**Table 4:** Crude and Adjusted Hazard ratio (95% CI) of 5-years cardiovascular mortality for Depression and Health Anxiety.

Variables	Crude HR (95% CI)	Model 1 HR (95% CI)	Model 2 HR (95% CI)	Model 3 HR (95% CI)
Depression/Health Anxiety Status				
No depression & No health anxiety	1 (Ref.)	1 (Ref.)	1 (Ref.)	1 (Ref.)
Only Depression	1.65 (1.45-2.34)	1.98 (1.75-2.21)	1.80 (1.59-2.89)	2.45 (1.84 - 3.03)
Only Health Anxiety^a^	N/A	N/A	N/A	N/A
Depression & Health Anxiety	1.05 (1.01-1.09)	1.06 (0.99-1.45)	1.13 (1.05-1.25)	2.11 (0.11 - 4.09)

**Model 1:** Adjusted for Demographic characteristics.

**Model 2:** Model 1 + Adjusted for Past Medical history.

**Model 3:** Model 2 + Adjusted for clinical data, (Full adjusted).

a N/A: Not applicable due to zero patients in this category (n = 0).

### 4.5 Model adjustments

• Model 1: Age, sex
• Model 2: Model 1 + smoking, diabetes, hypertension, hypercholesterolemia
• Model 3: Model 2 + ejection fraction, Killip class, MI location, number of affected vessels

When incorporating the composite psychological variable into the fully adjusted model, the ‘Health anxiety only’ category yielded no hazard estimate (N/A) due to a lack of patients in this group (n = 0). Compared to patients with neither condition, the risk of mortality was significantly elevated for individuals presenting with depression only (HR: 2.45, 95% CI: 1.84−3.03). Furthermore, individuals presenting with both depression and health anxiety exhibited an approximately two-fold increased mortality risk compared to the reference group (HR: 2.11, 95% CI: 0.11−4.09).

Proportional hazards assumption was evaluated using Schoenfeld residuals; no major violations were detected. Patients lost to follow-up were censored at last contact.

## 5 Summary

In this cohort, baseline depression was associated with increased 5-year cardiovascular mortality after adjustment for available covariates. Health anxiety showed a modest association in adjusted models. All associations are presented as adjusted associations, not causal effects. Limitations regarding measurement of health anxiety and potential residual confounding are described in the Discussion.

## 6 Discussion

The current study aimed to investigate the association of baseline depression and health anxiety with five-year cardiovascular mortality among patients with STEMI in the SEMI−CI cohort. Depression and health anxiety were associated with mortality, with depression showing a stronger association (Table 1, Table 2, Table 3, Table 4). These findings highlight the potential importance of identifying psychological symptoms in post-MI patients, without implying causality.

Consistent with previous studies, patients with health anxiety had higher observed long-term mortality rates [11]. This association persisted after adjustment for available demographic and clinical variables (Table 4). While the relationship between depression and myocardial infarction (MI) has been studied extensively, anxiety post-MI has received comparatively limited attention. This gap is mainly considering the high comorbidity between anxiety and depression [30]. To date, only a small number of prospective studies have investigated anxiety in the post-MI context, that showed inconsistent findings [[31], [32], [33]]. For instance, A nationwide registry study identified anxiety has been linked to a higher likelihood of mortality and recurrent cardiovascular events among individuals experiencing their first myocardial infarction [34], whereas other studies reported no such mortality association [32,33,35]. Another study offered mixed results, indicating that anxiety predicted cardiac events but not mortality [31]. In light of the limited research in this area, an observational work, provides this valuable contribution [36]. This association may occur through mechanisms like autonomic nervous system dysregulation, decreased physical activity, and poor adherence to treatment [11].

In our cohort, patients with health anxiety had a higher prevalence of smoking compared to those without health anxiety (Table 2). Few studies have examined this association in detail; such as an Australian study, noted that individuals with health anxiety are approximately twice as likely to be current smokers. In our cohort, we observed a similar trend, with 50.0% of patients in the health anxiety group being current smokers compared to 40.5% in the non-anxiety group [37]. Patients in the health anxiety group also showed a higher prevalence of hypercholesterolemia. According to our literature search, no studies to date have specifically examined this association or explored potential underlying mechanisms in this patient population [38].

Variability in previous study outcomes may be explained by differences in sample characteristics; for example, some studies included patients with arrhythmias or significant left ventricular dysfunction, populations at inherently higher risk of mortality [35,39]. Timing of anxiety assessment and the use of different diagnostic tools may have contributed to inconsistencies across studies. Additionally, the use of diverse assessment tools and diagnostic measures for anxiety may have introduced inconsistencies in the reported outcomes. Methodological limitations, including small sample sizes and limited adjustment for confounders, may affect the reliability of previous findings [39].

In our cohort, all patients with health anxiety also had depression, consistent with the high comorbidity reported in previous research. After MI, health anxiety may co-occur with depression, potentially contributing to psychological distress, reduced physical activity, and lower quality of life [40]. Prior mental health disorders, particularly previous anxiety episodes, and hospital complications have been associated with higher risk of post-MI depression [41].

Previous studies have reported an association between depression and increased mortality after MI. In this regard, studies from various countries investigated this effect in a large sample size, providing further evidence of the association [42]. Given the rising prevalence of depression among cardiac patients, it is essential to explore this relationship in developing countries as well [43]. Recent studies in the MENA (Middle East and North Africa) region have shown a high prevalence of depression among patients with MI; however, these studies have primarily focused on short-term mortality outcomes. Although a few studies conducted in these regions have noted similar effects on short-term prognosis, research examining the long-term implications of depression on cardiac outcomes remains limited [44,45].

One of the notable characteristics observed among individuals in the depression group was a higher prevalence of smoking. Large multicenter investigations conducted in Europe and Israel, have identified a link between post-MI depression and smoking behavior [46,47]. However, there has been a lack of large-scale, population-based data from the middle-income countries. This association may be partly explained by the use of smoking as a coping mechanism to alleviate psychological distress [48]. Furthermore, depressed patients had a higher prevalence of hypercholesterolemia (Table 3). As demonstrated in recent studies, Individuals with depression tend to participate less in behaviors that support cardiovascular health after experiencing a myocardial infarction. This includes lower adherence to a balanced diet, reduced levels of physical activity, poor compliance with prescribed medications, limited engagement in stress management practices, and lower completion rates of cardiac rehabilitation programs. Additionally, these patients often face greater challenges in achieving optimal cholesterol levels post-MI [49]. There were no significant differences between the two groups regarding other clinical and demographic data.

Strengths and Limitations:

The present study has several strengths, including its longitudinal cohort design and the focus on specific psychological factors in post-myocardial infarction patients. Although the observational design of our study precludes establishing a causal relationship, identifying these psychological factors as strong prognostic markers holds significant clinical value. From a clinical management perspective, these findings underscore the necessity of integrating routine psychological screening into post-MI care. Recognizing elevated depression or health anxiety can alert clinicians to a highly vulnerable subpopulation.

However, our study also has several limitations that should be acknowledged. First, due to the lack of systematic recording of non-fatal events, we could not evaluate Major Adverse Cardiovascular Events (MACE) and restricted our primary outcome to the post-myocardial infarction mortality rate. Second, as this was an observational cohort study utilizing all available data, an a priori sample size and power calculation was not performed. Third, we utilized a complete-case analysis approach for missing data, which may reduce statistical power.

Furthermore, while introducing the four-level composite variable effectively addressed the issue of multicollinearity, the hazard ratio for the “Both depression and health anxiety” category (HR: 2.11, 95% CI: 0.11-4.09) exhibited an exceptionally wide confidence interval. This imprecision is primarily driven by the small number of patients falling into this specific subgroup. Therefore, we urge readers to interpret this particular estimate with caution. The statistical reliability of this category is limited, and it should not be viewed with the same level of precision as the depression-only category.

Also, we did not have access to data regarding formal psychiatric diagnoses prior to admission, nor did we systematically record information on psychiatric treatments such as psychotropic medications or psychotherapy during the follow-up period. It remains unclear how these interventions might modify the observed associations, which highlights an important area for future research. Finally, our analysis is constrained by unmeasured confounders. We lacked data on critical factors such as lifestyle habits (diet and physical activity) and adherence to cardiovascular medications, all of which could significantly influence survival outcomes.

## 7 Conclusion

Given the high prevalence of depression and anxiety among post-MI patients, it is important to examine their association with long-term outcomes. Early identification of psychological symptoms may help guide supportive care and improve quality of life, but further research is needed to clarify causal relationships.

## Funding disclosures

This research did not receive any specific grant from funding agencies in the public, commercial, or not-for-profit sectors.

## CRediT authorship contribution statement

**Yasaman Shojaei:** Data curation, Formal analysis, Project administration, Writing – original draft, Writing – review & editing. **Hamidreza Roohafza:** Conceptualization, Supervision, Validation, Visualization. **Kamran Mehrabani-Zeinabad:** Methodology, Software. **Azam Soleimani:** Supervision, Validation. **Safoura Yazdekhasti:** Data curation, Software. **Mohammadamin Sadri:** Writing – original draft, Writing – review & editing. **Masoumeh Sadeghi:** Conceptualization, Investigation, Project administration, Supervision, Validation.

## Declaration of competing interest

The authors declare that they have no conflicts of interest regarding the publication of this paper.
